# Sleep in myalgic encephalomyelitis/chronic fatigue syndrome shows marked night-to-night fluctuation under free-living conditions—results from a matched case-control study

**DOI:** 10.1007/s44470-026-00079-7

**Published:** 2026-05-13

**Authors:** Maïtena Saurel, Isabelle Fornasieri, Giovanna C. Del Sordo, Cyril Chatain, Maria Livia Fantini, Mathieu Gruet, Oussama Saidi

**Affiliations:** 1 Laboratory Youth-Physical Activity, Sport and Health (J-AP2S), University of Toulon, CS 60584, 83041, Cedex 9 Toulon, France; 2 ASFC, French ME/CFS Association, Nice, France; 3 Faculty of Psychology, University of Strasbourg, Strasbourg, France; 4 Psychology Department, New Mexico State University, 1780 E University Blvd, Las Cruces, NM 88003 USA; 5 Inserm, CAPS UMR 1093, Burgundy Europe University, 21000 Dijon, France; 6 Sleep Disorders Center, Neurophysiology Unit, Neurology Department, Clermont-Ferrand University Hospital, Université Clermont Auvergne, CNRS, Institut Pascal, 58, Rue Montalembert, 63000 Clermont-Ferrand, France

**Keywords:** Intraindividual variability, Sleep irregularity, Sleep variability, Accelerometry, Sleepiness, Long covid

## Abstract

**Purpose:**

Unrefreshing and non-restorative sleep is a hallmark complaint in people with myalgic encephalomyelitis/chronic fatigue syndrome (ME/CFS). However, little is known about their habitual sleep and night-to-night fluctuations under real-life conditions. This study aimed to characterize sleep, and the intraindividual variability (IIV) of sleep in people living with ME/CFS compared with matched controls.

**Methods:**

In this case-control study, 38 ME/CFS and 38 controls wore a wrist accelerometer continuously for 7 days and completed concurrent sleep diaries, the Pittsburgh Sleep Quality Index (PSQI), and Epworth Sleepiness Scale (ESS). Within the ME/CFS group, participants were also stratified by symptom severity using the Bell Disability Scale. Sleep IIV was quantified using the coefficient of variation, the root mean square of successive differences, and the Bayesian variability model, respectively.

**Results:**

Compared with controls, individuals with ME/CFS spent significantly more time in bed and exhibited poorer sleep efficiency (SE) (all *p* < 0.05). Despite a longer time in bed, total sleep time did not differ between groups. ME/CFS participants also displayed significantly greater IIV in SE. By contrast, sleep timing (bedtime) was more regular among ME/CFS. Exploratory analyses did not detect clear differences across ME/CFS severity subgroups for mean sleep variables or variability indices.

**Conclusion:**

Under real-life conditions, people with ME/CFS exhibit poor sleep quality and unstable SE. These findings highlight sleep IIV as a clinically relevant dimension of sleep health in ME/CFS.

**Brief summary:**

**Current knowledge/study rationale:**

Unrefreshing sleep is a core symptom of ME/CFS, yet most evidence relies on single- or two-night laboratory assessments that may not reflect habitual sleep under real-life conditions. Moreover, night-to-night sleep variability, a potentially critical dimension of sleep health, has not been systematically examined in ME/CFS.

**Study impact:**

Using week-long wrist accelerometry, this study shows that under free-living conditions sleep in ME/CFS is characterized not only by impaired sleep efficiency but also by pronounced night-to-night variability, despite relatively stable bedtime compared to controls. These findings highlight sleep efficiency variability as a clinically relevant feature of ME/CFS and underscore the need for multi-night assessment and targeted strategies addressing sleep variability.

**Supplementary Information:**

The online version contains supplementary material available at 10.1007/s44470-026-00079-7.

## Introduction

Myalgic encephalomyelitis/chronic fatigue syndrome (ME/CFS) is a complex, multifactorial condition characterized by persistent fatigue and a variety of symptoms that drastically decrease daily activity levels and quality of life [1, 2]. Although the physiopathology of this chronic condition remains largely unknown, it appears to involve widespread abnormalities affecting the central nervous system, the immune system, and the endocrine system, as well as metabolism [3–5]. The true extent of ME/CFS global impact remains debated; however, it is estimated to affect between 10 and 70 million individuals worldwide, exceeding the prevalence of conditions such as multiple sclerosis or lupus [6, 7]. ME/CFS is frequently triggered by several viral infections, such as Epstein-Barr virus and other herpes viruses [8]. Recently, SARS-CoV-2 has been identified as a potential trigger, with nearly half of individuals with long COVID meeting the clinical diagnostic criteria for ME/CFS [9, 10]. This overlap has given rise to a resurgence of interest in ME/CFS, pointing out the necessity for a more profound comprehension of its symptoms, and the development of more effective management strategies [11].

As outlined in the 2015 Institute of Medicine (IOM) diagnostic criteria, unrefreshing sleep is a key symptom of ME/CFS [12]. A recent systematic review and meta-analysis reported increased time in bed (TIB) and total sleep time (TST) in people with ME/CFS compared with their healthy counterparts [13]. Despite the prolonged sleep duration, people with ME/CFS exhibited altered sleep quality, as indicated by decreased sleep efficiency (SE), increased sleep onset latency (SOL), and wake after sleep onset (WASO). Abnormalities in sleep staging were also reported, with a significant reduction in N2 and an increase in N3 sleep proportion. Although the reduction in rapid eye movement (REM) sleep did not reach significance, the meta-analysis documented a valuable increase in REM sleep latency. The majority of studies in this area were based on polysomnographic sleep assessment (PSG) (16 out of 20 studies in adults). Although PSG remains the gold standard strategy for diagnosing primary sleep disorders and examining sleep architecture, one or two nights of sleep assessment may not be representative of habitual sleep and may not provide information on all sleep health dimensions. In a correspondence addressed to Mohamed et al.’ s study, we discussed two key points for further consideration in future studies: inter- and intraindividual variability in sleep among ME/CFS [14]. In other words, are sleep disturbances a “one size fits all” ME/CFS? Are they consistent over time? Indeed, people living with ME/CFS form a heterogenous group in terms of disease severity, symptoms range, and intensity, as well as the context in which the disease develops. More importantly, like several other symptoms, sleep disturbances in ME/CFS can vary within the same individual and fluctuate over time. Therefore, PSG assessment, over 1 or 2 days may not always capture the full extent of sleep disturbances. Moreover, taking part in a laboratory sleep assessment may not be possible when patients experience increased symptoms. This could introduce a bias in the evaluation of sleep disturbances within this population [14].

Only a few studies have assessed sleep in people with ME/CFS in their home environment under free-living conditions. Available evidence suggests increased TIB and lower estimated SE, together with longer SOL and increased WASO compared to healthy controls [15–18]. However, to our knowledge, no study has examined night-to-night sleep fluctuations or stratified sleep patterns by disease severity. The primary aim of this study was to compare habitual sleep, measured via accelerometry, between individuals with ME/CFS and matched healthy controls. We also evaluated sleep across ME/CFS severity subgroups to explore whether greater clinical severity corresponds to more pronounced sleep disturbance. Beyond mean sleep duration and quality, we quantified intraindividual variability (IIV), including temporal irregularity in sleep timing and night-to-night fluctuations in SE and related sleep parameters. Because most existing evidence relies on a single-night polysomnography assessment, we further tested whether one night of sleep measurement can reliably represent a patient’s SE. We hypothesized that individuals with ME/CFS would exhibit greater sleep disruption and increased IIV compared with controls.

## Methods

### Study design and participants

This microlongitudinal matched case-control study reports results from the “IN-MOVEMENT” project implemented by the J-AP2S laboratory (University of Toulon, France), in collaboration with the French Association of Chronic Fatigue (www.asso-sfc.org, ASFC, Nice, France). Patients with ME/CFS and their matched healthy controls were recruited through e-mail correspondence delivered by both institutions. Pairs of groups were directly matched 1:1 by age (± 5 years), sex (male or female), and BMI (± 4 kg/m^2^). Eligible participants provided verbal and written consent and all were adults. Healthy controls were selected based on the absence of both past and present fatiguing conditions or primary sleep disorders, and were considered to be in good overall health. For eligibility, participants with ME/CFS were required to have a formal diagnosis established by a registered physician and to meet the International Consensus Criteria (ICC) [19]. Exclusion criteria were assessed through a structured interview conducted by a trained investigator, focusing on participants’ medical history. The main exclusion criteria were a diagnosed primary psychiatric disorder or a malignant disease within the past five years. Additional exclusion criteria included addictions and pregnancy. No formal objective diagnostic sleep assessments (e.g., polysomnography) were performed as part of the study protocol. However, participants reporting a diagnosed sleep disorder or a medical condition known to substantially affect sleep were excluded. Furthermore, individuals with ME/CFS with severe symptoms at rest or on a continuous basis according to the Bell Chronic Fatigue Syndrome (CFS) Disability Scale were also excluded from the study, as they were mainly bedridden and thus unable to manage and tolerate the data collection procedure. Ethical clearance for this study was obtained from an Institutional Review Board (IRB00012476-2024-2103–302, which ensured that all assessments adhered strictly to the ethical principles set out in the Declaration of Helsinki.

### Procedure

After enrollment, participants completed an interview to collect sociodemographic information (e.g., age, sex, employment status) and medical history, including prior infections, duration of ME/CFS symptoms, treatments, and comorbidities. Afterward, the follow-up was conducted in the participants’ habitual environments. Each participant received a package by mail containing detailed instructions, a wrist-worn accelerometer (GT3X+, ActiGraph LLC, Pensacola, FL, USA), and a booklet of questionnaires. The booklet included a sleep diary to be completed throughout the microlongitudinal follow-up [20], as well as the following questionnaires: the Pittsburgh Sleep Quality Index (PSQI) [21], and the Epworth Sleepiness Scale [22]. Furthermore, participants with ME/CFS were stratified into three groups based on their scores on the Bell CFS Disability Scale: moderate to severe (20–30), moderate (31–49), and mild to moderate (≥ 50), consistent with prior literature on ME/CFS functional classification [11, 23]. Upon completion of the follow-up, participants returned the package, by post to the laboratory.

### Measures

#### Sleep monitoring and analyses

Accelerometry provides a non-invasive, ecologically valid method for sleep assessment in real-life conditions. However, accelerometry does not directly measure sleep stages or architecture and should therefore be considered a proxy-based method rather than a diagnostic tool. Consequently, sleep parameters derived from accelerometry represent estimated indices of sleep-wake patterns and must be interpreted with appropriate caution. Raw triaxial data were collected using ActiGraph GT3X+ devices (ActiGraph LLC, Pensacola, FL, USA) worn continuously on the non-dominant wrist for seven consecutive days, excluding water-based activities, at a sampling rate of 60 Hz. Data were processed in R (version 4.0.5; R Core Team, 2021) using the open-source GGIR package (version.3) to estimate sleep parameters [24]. GGIR operates on raw acceleration data and internally aggregates to short epochs for analysis, applies automatic calibration and non-wear detection, and supports integration of sleep diaries. Sleep-wake classification relied on GGIR’s Heuristic Decision Tree algorithm validated against polysomnography in free-living settings [25]. Daily sleep diaries provided self-reported bed and wake times, which were used as anchors to refine detection of the main nocturnal sleep episode and exclude naps. Sleep diary data were used as temporal anchors to define bedtime and wake-up time windows for accelerometry scoring. Diary-reported lights-off and final awakening times guided the identification of total time in bed (TIB). When diary entries were available, accelerometry data were aligned with the diary reports. Discrepancies between diary and accelerometry estimates were uncommon and were resolved through visual inspection of the accelerometry signal (e.g., activity counts) to determine the most plausible TIB interval. When diary data were missing, TIB intervals were identified using accelerometry-based algorithms, complemented by visual inspection when needed. Diary completion was requested on a daily basis, and adherence to the study protocol was very high (≈98% of nights). Outputs were visually inspected for plausibility and artifacts (i.e., device non-wear). Extracted sleep estimated metrics included bedtime, wake-up time, TIB, TST, SE, WASO, and SOL.

#### Intraindividual variability (IIV) of sleep

IIV of sleep parameters was quantified using three complementary indices: the Coefficient of Variation (CV), the Root Mean Square of Successive Differences (RMSSD), and the Bayesian Variability Model (BVM) [26, 27]. These indices were selected because they capture distinct and complementary dimensions of night-to-night variability. All participants contributed the same number of nights, ensuring that variability estimates were not influenced by differences in data availability.

The Coefficient of Variation (CV) reflects the overall magnitude of variability relative to an individual’s mean level. In practical terms, CV indicates how large the fluctuations are compared with the typical value of the sleep parameter. Higher CV values therefore represent greater global dispersion across nights. For each participant, CV was calculated as the ratio of the within-person standard deviation to the mean value across all valid nights, expressed as a percentage. The RMSSD captures short-term instability by quantifying the average magnitude of changes between consecutive nights. Unlike CV, which summarizes overall dispersion, RMSSD is sensitive to the night-to-night irregularity of sleep patterns. Higher RMSSD values indicate greater sequential variability (i.e., less stability from one night to the next). Finally, the Bayesian Variability Model (BVM) was used to estimate latent (true) intraindividual variability by modeling nightly values as probabilistic deviations around each participant’s mean. This approach provides an estimate of within-person variance while explicitly accounting for measurement uncertainty. Conceptually, the BVM differs from conventional dispersion metrics (e.g., CV, RMSSD) because it separates systematic (latent) variability from residual variability. This distinction yields a more conservative and uncertainty-aware estimate of night-to-night instability. However, residual variability, although often treated as random error, may still reflect meaningful context-dependent or physiological fluctuations. For transparency, the mathematical specification of the IIV indices is provided in Table S1.

#### Questionnaires

- **Pittsburgh Sleep Quality Index (PSQI):** Subjective sleep quality was assessed using the PSQI, a validated 19-item questionnaire evaluating sleep over the past month [21]. It yields seven component scores (e.g., sleep latency, duration, disturbances), each rated 0–3. The sum produces a global score from 0 to 21, with scores > 5 indicating poor sleep quality.
- **Epworth sleepiness scale (ESS):** Daytime sleepiness was measured using the ESS, an 8-item self-report assessing general level of daytime sleepiness in common situations [22]. Each item is rated on a 0–3 scale, yielding a total score from 0 to 24; scores > 10 suggest excessive sleepiness.
- **Bell CFS Disability Scale:** The Bell CFS Disability Scale is a self-report tool used to assess functional impairment in individuals with ME/CFS. It ranges from 0 (severely disabled) to 100 (fully functional), with intermediate scores reflecting varying degrees of physical activity, work capacity, and symptom severity [23]. Despite its specificity to people with ME/CFS, this scale is not formally validated through standard psychometric procedures (e.g., construct validity, test-retest reliability, factor analysis) in peer-reviewed literature.

### Statistical analyses

The primary analysis compared people living with ME/CFS and matched healthy controls. With 38 participants per group and a two-sided α level of 0.05 for the primary analysis, the study provides approximately 80% power to detect standardized mean differences of about *d* ≈ 0.65, and > 90% power for effects ≥ 0.75. This sample size was therefore considered sufficient to detect moderate-to-large effects in the primary case-control comparisons. Secondary analyses stratified by disease severity were predefined as exploratory and were not powered for confirmatory inference. For the primary case-control analyses, *p*-values were used to aid statistical inference together with effect size estimates, whereas secondary analyses were interpreted descriptively, with emphasis on effect sizes and patterns of results rather than on statistical significance. Because the primary analyses addressed a single overarching research question and involved multiple highly correlated sleep outcomes derived from accelerometry, no formal correction for multiple comparisons was applied in order to limit inflation of type II error.

All analyses were performed in RStudio using R version 4.4.1 (R Core Team, 2024). Visualizations were generated with ggplot2, and figure assembly was conducted using ggpubr. Descriptive statistics are reported as mean ± standard deviation unless otherwise specified. Between-group comparisons (ME/CFS vs controls) for continuous variables were examined using independent-samples t-tests, and effect sizes were calculated as Cohen’s *d*. Comparisons across ME/CFS severity subgroups were performed using Kruskal-Wallis tests, with Eta^2^ reported as the effect size; when significant, Dunn post hoc tests with Holm correction were applied. Categorical variables were analyzed using Chi-square tests or Fisher’s exact tests, with effect sizes expressed as Cramer’s *V*. The same statistical approach (t-tests for two-group comparisons; Kruskal-Wallis tests for subgroup analyses) was applied to habitual sleep measures and intraindividual variability indices (CV, RMSSD, and BVM). As a sensitivity analysis, primary case-control comparisons were re-estimated using linear regression models including Group (ME/CFS vs controls) and employment status (binary: employed vs non-employed) as predictors to account for potential differences in daytime structure.

## Results

### Sample characteristics and subjective sleep

Thirty-eight individuals with ME/CFS and matched healthy controls were enrolled in this study. Sociodemographic, clinical, and subjective sleep characteristics are summarized in Table 1, and further information on comorbidities and ongoing treatments is provided in Table S2. Compared with healthy controls, people with ME/CFS reported poorer subjective sleep quality, with large effect sizes across all PSQI domains except sleep duration. Notably, 95% of participants with ME/CFS exceeded the clinical PSQI cutoff (> 5), indicating pervasive and clinically meaningful sleep disturbances compared to controls (*p* < 0.001). Daytime sleepiness, assessed by ESS, was also significantly elevated in the ME/CFS group relative to their healthy counterparts (*p* < 0.001) with 47% showing excessive daytime sleepiness. Subjective sleep disturbances were consistently observed across all disease severity subgroups. However, the proportion of participants with excessive sleepiness (ESS > 10) did differ significantly across severity levels (*p* = 0.014, large effect size = 0.48).

**Table 1:** Demographics, clinical, and subjective sleep related characteristics among people with myalgic encephalomyelitis/chronic fatigue syndrome (ME/CFS) including severity subgroups compared to healthy controls

	Controls	ME/CFS			ME/CFS [Mild to Moderate]	ME/CFS [Moderate]	ME/CFS [Moderate to Severe]		
	(*N* = 38)	(*N* = 38)	*p*	Effect Size [Interpretation]	(*N* = 5)	(*N* = 20)	(*N* = 13)	*p*	Effect Size [Interpretation]
**Demographic data**									
Age (years)	48 ± 11.88	51 ± 10.13	0.240	0.27 [Small]	57 ± 10.84	53.7 ± 10.09	44.54 ± 6.60	**0.003**	0.28 [Large]
BMI (kg/m^2^)	23.68 ± 4.01	23.03 ± 4.44	0.500	0.16 [Small]	22.60 ± 5.46	24 ± 4.94	21.69 ± 2.93	0.508	0.02 [Negligible]
Sex			1.000	0 [Negligible]				0.346	0.26 [Medium]
Female (*N*, %)	30, 78.95%	30, 78.95%			5, 100%	14, 70%	11, 84.62%		
Male (*N*, %)	8, 21.95%	8, 21.95%			0, 0%	6, 30%	2, 15.38%		
Employment status			** < 0.001**	0.71 [Large]				**0.019**	0.48 [Large]
Employed (*N*,%)	27, 71.05%	6, 15.79%			3, 60%	1, 5%	2, 15.38%		
Unemployed (*N*, %)	5, 13.16%	3, 7.89%			0, 0%	3, 15%	0, 0%		
Retired (*N*, %)	2, 5.26%	5, 13.16%			1, 20%	4, 20%	0, 0%		
Unable to work (*N*, %)	0, 0%	22, 57.89%			1, 20%	10, 50%	11, 84.62%		
Homemaker (*N*, %)	4, 10.53%	2, 5.26%			0, 0%	2, 10%	0, 0%		
**Clinical data**									
Duration of illness (months)	N/A	129.90 ± 123.99			134.4 ± 81.56	138 ± 139.70	87 ± 73.28	0.377	< 0.01 [Negligible]
Diagnostic delay (months)	N/A	34.71 ± 46.71			27.8 ± 31.63	49 ± 60.36	20.46 ± 21.60	0.239	0.02 [Negligible]
Bell CFS Disability Scale	N/A	40.54 ± 10.53			58.75 ± 2.50	44.25 ± 5.68	29.23 ± 2.77	** < 0.001**	0.84 [Large]
**Subjective sleep-related data**									
PSQI global score									
Subjective sleep quality	0.45 ± 0.50	1.63 ± 0.71	** < 0.001**	1.92 [Large]	1.8 ± 0.84	1.5 ± 0.69	1.77 ± 0.73	0.681	0.04 [Negligible]
Sleep latency	1.21 ± 0.58	1.76 ± 0.94	**0.003**	0.71 [Large]	1.6 ± 1.14	1.85 ± 0.99	1.69 ± 0.85	0.846	0.05 [Negligible]
Sleep duration	0.42 ± 0.50	0.45 ± 0.76	0.859	0.04 [Negligible]	0.80 ± 1.30	0.45 ± 0.69	0.31 ± 0.63	0.667	0.03 [Negligible]
Sleep efficiency	0.47 ± 0.51	1.35 ± 1.34	** < 0.001**	0.87 [Large]	1.6 ± 1.52	1.45 ± 1.32	1.08 ± 1.38	0.622	0.03 [Negligible]
Sleep disturbance	0.45 ± 0.50	1.76 ± 0.54	** < 0.001**	2.51 [Large]	1.6 ± 0.55	1.75 ± 0.55	1.85 ± 0.55	0.694	0.04 [Negligible]
Need for sleep inducers	0.03 ± 1.16	0.87 ± 1.32	** < 0.001**	0.90 [Large]	1.6 ± 1.52	0.7 ± 1.26	0.85 ± 1.34	0.383	0.002 [Negligible]
Daytime dysfunction	0.08 ± 0.27	1.55 ± 0.92	** < 0.001**	2.17 [Large]	1.4 ± 0.89	1.45 ± 0.89	1.77 ± 1.01	0.462	0.01 [Negligible]
Total	3.11 ± 0.39	9.37 ± 3.17	** < 0.001**	2.78 [Large]	10.40 ± 4.16	9.15 ± 3.34	9.31 ± 2.63	0.771	0.04 [Negligible]
Score > 5 (*N*, %)	0, 0%	35, 94.59%	** < 0.001**	0.92 [Large]	5, 100%	17, 89.47%	13, 100%	0.629	0.23 [Medium]
ESS score									
Score > 10 (*N*, %)	0, 0%	18, 47.38%	** < 0.001**	0.53 [Large]	4, 80%	5, 25%	9, 69.23%	**0.014**	0.48 [Large]
Total	4.45 ± 1.18	10.26 ± 6.52	**< 0.001**	1.24 [Large]	10.60 ± 6.54	8.50 ± 6.08	12.84 ± 6.77	0.140	0.06 [Small]

Data are presented as mean ± standard deviation or as number of participants and percentages. Duration of illness refers to the time since symptom onset, and diagnostic delay to the time until formal diagnosis. Employment categories represent self-reported current status

*BMI*, body mass index; *CFS*, chronic fatigue syndrome; *ESS*, Epworth Sleepiness Scale; *ME/CFS*, myalgic encephalomyelitis/chronic fatigue syndrome; *N*, number of participants; *N/A*, not applicable; *PSQI*, Pittsburgh Sleep Quality Index; *SD*, standard deviation. Bold values indicate statistically significant differences between groups (*p*<0.05)

### Comparison of habitual sleep between ME/CFS Patients and Healthy Controls (including severity subgroups)

As shown in Table 2, compared with healthy controls, individuals with ME/CFS did not differ significantly in bedtime (*p* = 0.053, ES = 0.45 [Medium effect]), later morning wake times (*p* < 0.05, ES = 0.46 [Medium effect]), resulting in significantly longer TIB (*p* < 0.001). However, ME/CFS showed reduced accelerometry estimated SE (*p* < 0.001), marked by a nearly doubled sleep onset latency (*p* < 0.001) and increased wake after sleep onset (*p* < 0.001). Despite spending nearly an extra hour in bed, TST did not differ between the two groups. Sleep disturbances appeared broadly similar across severity subgroups, with negligible effect sizes observed between strata.

**Table 2:** Habitual sleep among people with myalgic encephalomyelitis/chronic fatigue syndrome (including severity subgroups) compared to healthy controls

	Controls	ME/CFS			ME/CFS [Mild to Moderate]	ME/CFS [Moderate]	ME/CFS [Moderate to Severe]		
	(*N* = 38)	(*N* = 38)	*p*	Effect Size [Interpretation]	(*N* = 5)	(*N* = 20)	(*N* = 13)	*p*	Effect Size [Interpretation]
Bedtime (hh:mm ± hh:min)	23:06 ± 00:25	22:44 ± 01:05	0.053	0.45 [Medium]	22:01 ± 00:34	22:43 ± 00:57	23:02 ± 01:20	0.125	0.06 [Small]
Wake-up time (hh:mm ± hh:min)	07:18 ± 00:23	07:44 ± 01:15	**0.048**	0.46 [Medium]	07:15 ± 00:28	07:38 ± 01:29	08:04 ± 00:59	0.125	0.06 [Small]
TIB (hh:mm ± min)	08:11 ± 00:35	08:59 ± 01:15	** < 0.001**	0.80 [Large]	09:13 ± 00:47	08:55 ± 01:24	09:01 ± 01:16	0.871	0.05 [Small]
TIB (min)	491.58 ± 35.99	539.32 ± 75.77	** < 0.001**	0.80 [Large]	553.4 ± 46.84	534.9 ± 83.69	540.69 ± 75.91	0.871	0.05 [Small]
TST (hh:mm ± min)	06:48 ± 00:31	06:55 ± 00:53	0.491	0.16 [Small]	07:00 ± 00:49	06:52 ± 00:58	06:57 ± 00:49	0.950	0.05 [Small]
TST (min)	407.68 ± 31.08	414.58 ± 52.90	0.491	0.16 [Small]	420 ± 48.63	411.90 ± 58.31	416.62 ± 49.27	0.950	0.05 [Small]
SE (%)	82.5 ± 1.89	76.71 ± 3.55	** < 0.001**	2.04 [Large]	75.40 ± 2.70	76.85 ± 3.54	77 ± 3.96	0.749	0.04 [Small]
SOL (min)	19.05 ± 7.01	33.21 ± 12.88	** < 0.001**	1.37 [Large]	29.20 ± 3.42	31.30 ± 12.68	37.69 ± 7.01	0.381	0.001 [Negligible]
WASO (min)	63.82 ± 13.75	90.79 ± 26.93	** < 0.001**	1.26 [Large]	103.60 ± 11.57	91.20 ± 26.51	85.23 ± 31.25	0.171	0.04 [Small]

Data are presented as mean ± standard deviation. Sleep parameters were obtained from one week of continuous wrist-worn accelerometry. Bedtime and wake-up time represent the average timing of nocturnal sleep episodes. Total time in bed refers to the duration between bedtime and final wake-up time, while total sleep time represents the estimated amount of sleep within this period. Sleep efficiency is calculated as the ratio of total sleep time to total time in bed, expressed as a percentage. Sleep onset latency indicates the time required to transition from wakefulness to sleep, and wake after sleep onset reflects the total duration of awakenings occurring after sleep onset

*ME/CFS*, myalgic encephalomyelitis/chronic fatigue syndrome; *SD*, standard deviation; *SE*, Sleep Efficiency; *SOL*, Sleep Onset Latency; *TIB*, Time in Bed; *TST*, Total Sleep Time; *WASO*, Wake After Sleep Onset. Bold values indicate statistically significant differences between groups (*p*<0.05)

### Intraindividual variability in sleep between ME/CFS and healthy controls (including severity subgroups)

IIV of sleep including temporal instability of sleep timing and night-to-night fluctuations in sleep metrics are presented in Table 3.

**Table 3:** Intraindividual variability of sleep among people with myalgic encephalomyelitis/chronic fatigue syndrome (ME/CFS) including severity subgroups compared to healthy controls

	Controls	ME/CFS			ME/CFS [Mild to Moderate]	ME/CFS [Moderate]	ME/CFS [Moderate to Severe]		
	Mean ± SD (*N* = 38)	Mean ± SD (*N* = 38)	*p*	Effect Size [Interpretation]	Mean ± SD (*N* = 5)	Mean ± SD (*N* = 20)	Mean ± SD (*N* = 13)	*p*	Effect Size [Interpretation]
**CV**									
Bedtime (%)	2.97 ± 0.72	2.32 ± 1.44	**0.014**	0.58 [Medium]	2 ± 0.71	2.5 ± 1.67	2.15 ± 1.28	0.823	0.05 [Small]
Wake-up time (%)	9.05 ± 1.75	9.58 ± 5.45	0.573	0.13 [Small]	8.8 ± 4.55	9.45 ± 5.33	10.08 ± 6.26	0.927	0.05 [Small]
TIB (%)	11.13 ± 3.73	9.13 ± 4.87	**0.048**	0.46 [Medium]	7 ± 1.58	9.65 ± 5.19	9.15 ± 3.73	0.513	0.02 [Negligible]
TST (%)	11.29 ± 3.73	13.87 ± 4.70	**0.010**	0.61 [Medium]	12.40 ± 3.58	13.6 ± 3.38	14.85 ± 6.61	0.559	0.02 [Negligible]
SE (%)	2.53 ± 1.03	10.13 ± 2.77	** < 0.001**	3.64 [Large]	10.6 ± 1.52	9.7 ± 2.70	10.62 ± 3.28	0.405	0.01 [Negligible]
SOL (%)	57.29 ± 25.52	59.18 ± 24.61	0.743	0.08 [Negligible]	53.80 ± 13.61	57.80 ± 21.19	63.38 ± 32.56	0.961	0.05 [Small]
WASO (%)	29.24 ± 14.47	43.92 ± 16.67	** < 0.001**	0.94 [Large]	38.20 ± 2.49	44.45 ± 16.50	45.31 ± 20.18	0.481	0.02 [Negligible]
**RMSSD**									
Bedtime (hh:mm ± hh:min)	01:00 ± 00:24	00:48 ± 00:26	**0.045**	0.47 [Medium]	00:32 ± 00:10	00:52 ± 00:30	00:49 ± 00:23	0.319	0.01 [Negligible]
Wake-up time (hh:mm ± hh:min)	00:41 ± 00:07	01:01 ± 00:40	**0.004**	0.69 [Large]	00:58 ± 00:28	00:56 ± 00:35	01:10 ± 00:52	0.815	0.05 [Small]
TIB (min)	76.74 ± 26.41	74.71 ± 43.53	0.807	0.06 [Negligible]	59.80 ± 17.81	74 ± 37.21	81.54 ± 53.31	0.673	0.03 [Negligible]
TST (min)	65.24 ± 23.36	80.05 ± 34.35	**0.031**	0.50 [Medium]	80.40 ± 31.67	76.25 ± 26.97	85.77 ± 45.76	0.926	0.05 [Small]
SE (%)	2.66 ± 1.21	10.32 ± 3.87	** < 0.001**	2.67 [Large]	11.60 ± 2.30	9.65 ± 3.98	10.85 ± 4.20	0.444	0.01 [Negligible]
SOL (min)	15.26 ± 9.93	27.39 ± 17.75	** < 0.001**	0.84 [Large]	21.40 ± 5.77	24.85 ± 17.02	33.62 ± 20.82	0.486	0.02 [Negligible]
WASO (min)	26.29 ± 12.08	49.03 ± 21.64	** < 0.001**	1.30 [Large]	56.20 ± 19.58	48.35 ± 22.11	47.31 ± 22.72	0.702	0.04 [Negligible]
**BVM**									
Bedtime (a.u.)	64.47 ± 9.48	60.16 ± 8.31	**0.038**	0.48 [Medium]	58 ± 9.82	60 ± 8.18	61.23 ± 8.47	0.677	0.03 [Negligible]
Wake-up time (a.u.)	58.66 ± 29.33	60 ± 31.18	0.847	0.04 [Negligible]	77.20 ± 3.03	57.15 ± 29.80	57.77 ± 32.94	0.361	0.001 [Negligible]
TIB (a.u.)	80.61 ± 16.17	77.03 ± 15.62	0.330	0.23 [Small]	80.61 ± 19.59	76.27 ± 12.99	78.44 ± 18.74	0.897	0.05 [Small]
TST (a.u.)	64.92 ± 9.55	63.71 ± 9.16	0.574	0.13 [Small]	66.20 ± 9.96	62.95 ± 7.87	65.44 ± 10.97	0.703	0.04 [Negligible]
SE (a.u.)	5.68 ± 2.47	5.66 ± 2.54	0.964	0.01 [Negligible]	5.52 ± 2.26	6.27 ± 2.85	6.14 ± 2.35	0.946	0.05 [Small]
SOL (a.u.)	19.05 ± 7.59	17.50 ± 8.72	0.410	0.19 [Small]	19.40 ± 16.68	18.70 ± 8.52	14.92 ± 3.95	0.563	0.02 [Negligible]
WASO (a.u.)	36.05 ± 10.03	37.84 ± 10.04	0.440	0.18 [Small]	35 ± 9.27	38.35 ± 11.54	38.15 ± 8.20	0.781	0.04 [Negligible]

Data are presented as mean ± standard deviation. Intraindividual variability (IIV) of sleep was calculated across one week of accelerometry recordings. CV expresses relative variability as a percentage. RMSSD quantifies short-term night-to-night fluctuations and is expressed in the same units as the corresponding parameter. BVM provides a standardized, unitless estimate of true within-person variance after accounting for measurement error and uncertainty

*a.u.*, arbitrary units; *BVM*, Bayesian Variability Model; *CV*, coefficient of variation; *ME/CFS*, myalgic encephalomyelitis/chronic fatigue syndrome; *RMSSD*, root mean square of successive differences; *SD*, standard deviation; *SE*, sleep efficiency; *SOL*, sleep onset latency; *TIB*, total time in bed; *TST*, total sleep time; *WASO*, wake after sleep onset. Bold values indicate statistically significant differences between groups (*p*<0.05)

#### Coefficient of variation (CV)

Compared with healthy controls, individuals with ME/CFS showed significantly higher variability in estimated sleep metrics including duration, and continuity, as indicated by increased CV for TST (*p* = 0.010, ES = 0.61 [Medium]), SE (*p* < 0.001, ES = 3.64 [Large]), and WASO (*p* < 0.001, ES = 0.94 [Large]). In contrast, variability in bedtime and TIB was lower in ME/CFS (*p* = 0.014, ES = 0.58 [Medium]; *p* = 0.048, ES = 0.46 [Medium], respectively), while no group differences were observed for wake-up time or SOL (all *p* > 0.05, ES = 0.08–0.13). Across ME/CFS severity subgroups, exploratory comparisons did not reveal clear differences in CV parameters, with observed effect sizes remaining negligible to small.

#### Root mean square of successive differences (RMSSD)

ME/CFS patients displayed greater night-to-night variability in both sleep initiation and continuity, reflected by higher RMSSD values for TST (*p* = 0.031, ES = 0.50 [Medium]), SE (*p* < 0.001, ES = 2.67 [Large]), SOL (*p* < 0.001, ES = 0.84 [Large]), and WASO (*p* < 0.001, ES = 1.30 [Large]). Wake-up time instability was also elevated in ME/CFS compared to healthy controls (*p* = 0.004, ES = 0.69 [Large]), whereas bedtime instability was lower (*p* = 0.045, ES = 0.47 [Medium]), and TIB showed no substantial group differences (ME/CFS vs. controls; *p* > 0.05). Similar to CV results, no RMSSD clear differences emerged between ME/CFS severity subgroups (ES = negligible–small).

#### Bayesian variability model (BVM)

BVM estimates revealed no significant differences between groups for wake-up time, TIB, TST, SE, SOL, or WASO (all *p* > 0.33, ES = negligible–small). Only bedtime variability remained lower in ME/CFS compared to controls (*p* = 0.038, ES = 0.48 [Medium]). No between-severity differences were obvious for any BVM metric (all ES = negligible).

#### Comparison of intraindividual variability metrics results

Analyses of intraindividual variability revealed distinct patterns depending on the variability metric applied. Both CV and RMSSD consistently indicated greater night-to-night fluctuation in several sleep parameters among individuals with ME/CFS compared with controls. These indices, which capture overall dispersion across nights (CV) and short-term successive fluctuations (RMSSD), suggest that sleep in ME/CFS is characterized by higher variability. In contrast, BVM, which isolates stable intraindividual variance while accounting for measurement uncertainty, did not identify corresponding group differences, indicating the absence of a structured or systematic pattern of variability. This suggests that the instability detected by conventional indices may reflect irregular or context-dependent fluctuations rather than consistent within-person variance. Notably, BVM-derived variability in bedtime remained significantly lower in the ME/CFS group compared with controls (*p* = 0.038), consistent with reduced variability in sleep timing previously reported using CV and RMSSD.

### Sensitivity analyses

In sensitivity analyses adjusting for employment status, the primary case-control differences in estimated SE, SOL, WASO, and key variability indices remained similar. Employment status was not independently associated with the sleep outcomes (Table S3).

### Estimated sleep efficiency variability in ME/CFS challenges single-night evaluation

**Fig. 1:**
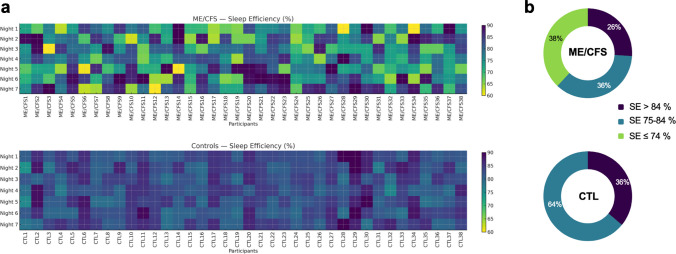
Sleep efficiency patterns and night-category distributions in myalgic encephalomyelitis/chronic fatigue syndrome (ME/CFS) and controls. **a** Individual heatmaps showing sleep efficiency across seven consecutive nights in participants with ME/CFS (top panel) and healthy controls (bottom panel). Each cell represents a single night per participant, with brighter colors indicating lower sleep efficiency and darker colors reflecting higher efficiency. **b** Proportion of nights falling into three sleep efficiency categories (< 74%, 74–85%, > 85%) for each group. SE categories were defined according to the National Sleep Foundation’s adult sleep quality recommendations, with SE > 85% considered appropriate, 74–85% uncertain, and < 74% inappropriate [44]. *CTL*, controls; *ME/CFS*, myalgic encephalomyelitis/chronic fatigue syndrome; *SE*, sleep efficiency

Figure 1a shows that estimated SE in ME/CFS exhibits pronounced night-to-night variability, with frequent alternations between low- and high-efficiency nights, whereas controls display much more stable patterns. As illustrated in Fig. 1b, this variability translates into a significantly different distribution of estimated SE categories between groups (χ^2^(2) = 128.5, *p* < 0.001), with a moderate-to-large effect size (Cramer’s *V* = 0.49). Consequently, a single-night assessment may fail to capture low-efficiency nights or may overrepresent high-efficiency nights, thereby underestimating the overall burden and variability of sleep disturbances in ME/CFS.

## Discussion

The present study provides a refined characterization of habitual sleep disturbances in ME/CFS under real-life conditions by moving beyond mean sleep parameters to explicitly examine night-to-night fluctuation. While individuals with ME/CFS spent more time in bed and exhibited lower estimated SE than matched controls, total sleep time did not differ between groups. This dissociation reinforces prior evidence that prolonged sleep opportunity in ME/CFS does not translate into restorative sleep, and suggests that the primary disturbance may lie in the capacity to initiate and maintain sleep rather than sleep duration [15–18]. Importantly, the current findings extend this literature by demonstrating that impaired estimated SE in ME/CFS is not only reduced on average but also highly unstable across nights, even in the presence of relatively regular sleep timing compared to healthy controls.

Sleep normally exhibits some degree of night-to-night fluctuation in response to environmental, psychosocial, and physiological influences [28]. However, the magnitude of variability observed in ME/CFS exceeded that reported in healthy populations and was comparable to levels documented in clinical sleep disorders such as insomnia [29–31]. For instance, using accelerometry, Veeramachaneni et al. reported a within-person standard deviation in estimated SE of 5.83% in young adults with insomnia, corresponding to a CV of approximately 7.0% [29]. Buysse et al. observed a slightly higher SE variability in older adults with chronic insomnia (SD = 7.0%; CV ≈ 8.6%) [30], and Chan et al. reported a within-person standard deviation of 6.7%, corresponding to an estimated CV of approximately 8–8.5% [31]. Notably, this heightened estimated SE variability occurred despite preserved or even reduced variability in bedtime, contrasting with insomnia populations in which irregular sleep timing often accompanies poor sleep continuity [29–31]. This dissociation challenges maladaptive sleep behaviors and may instead point toward instability within physiological systems governing sleep maintenance. Consistent with this interpretation, prior work has shown that excessive night-to-night variability in sleep continuity is associated with inflammatory processes, autonomic dysregulation, and altered homeostatic sleep pressure, independent of mean sleep duration or efficiency [32]. In ME/CFS, such mechanisms are highly plausible [33, 34]

A key contribution of the present study is the simultaneous application of complementary variability metrics. Conventional dispersion indices (CV and RMSSD) consistently indicated greater night-to-night fluctuations in estimated SE and related parameters in ME/CFS. In contrast, the Bayesian variability model, which isolates stable latent variability while accounting for measurement uncertainty, did not reveal corresponding group differences. This divergence may suggest that the observed variability may primarily reflect context-dependent fluctuations rather than a fixed, trait-like increase in underlying variability. Importantly, the residual component captured by CV and RMSSD may in fact encode physiologically meaningful responses to daily perturbations. In ME/CFS, such perturbations could include variations in symptom burden, physical or cognitive exertion, and post-exertional malaise (PEM). The finding of reduced bedtime variability alongside unstable SE further supports this interpretation, suggesting that individuals with ME/CFS may actively maintain regular sleep schedules as an adaptive pacing strategy, yet remain unable to achieve consistent sleep continuity due to fluctuating internal constraints. We therefore propose that pronounced night-to-night sleep variability in ME/CFS may partly reflect the characteristic “push-and-crash” dynamic of the illness. Periods of increased activity may precipitate delayed physiological deterioration, manifesting as PEM and subsequent sleep fragmentation [35, 36]. Analogous patterns have been observed during acute infection, where sleep duration may increase while continuity deteriorates as part of an inflammation-driven response [37]. In ME/CFS, even minor daily activities could trigger such responses, leading to unpredictable alternations between relatively preserved and markedly impaired nights of sleep. However, sleep under free-living conditions is also influenced by daytime structure and environmental context [32]. Although employment status differed markedly between groups, sensitivity analyses adjusting for employment status did not materially alter the primary findings, suggesting that the observed sleep disturbances and variability in ME/CFS are unlikely to be solely explained by differences in daytime occupational structure.

In addition, circadian disruption has been proposed as a contributing mechanism in ME/CFS, potentially involving altered coordination by the suprachiasmatic nucleus and downstream desynchronization of sleep-wake, autonomic, and metabolic processes [38]. Genetic and transcriptomic studies reported markedly elevated expression of the clock gene NPAS2 in ME/CFS patients, although these investigations remain underpowered and lack repeated rhythmic sampling [39]. Studies using accelerometry consistently describe reduced daytime activity, lower rhythm amplitude, and irregular rest-activity patterns. However, interpretation remains limited by masking effects and the absence of constant routine or forced desynchrony protocols capable of isolating endogenous circadian signals [40]. In addition, lifestyle constraints frequently observed in ME/CFS, including lower employment rates and increased time spent indoors, may reduce exposure to light, a primary circadian zeitgeber, thereby potentially exacerbating circadian misalignment. Physiological markers show mixed findings, with largely preserved mean temperature and melatonin timing but altered coupling between dim light melatonin onset (DLMO) and temperature rhythms, increased variability, and atypical distal skin temperature profiles [16, 40]

Alongside this circadian dysregulation, the variability of sleep patterns becomes particularly relevant. In fact, large-scale studies suggest that irregular sleep patterns, rather than sleep duration alone, are more strongly associated with adverse cardiovascular outcomes and circadian disruption [41, 42]. The present findings are particularly noteworthy, as they demonstrate marked night-to-night sleep variability in ME/CFS despite relatively preserved bedtime regularity, pointing to variability of sleep continuity as a distinct feature of the disorder. Recent data further indicate an elevated cardiovascular risk in ME/CFS, potentially driven by chronic low-grade inflammation, autonomic dysfunction, and endothelial impairment [43]. Whether sleep irregularity contributes to this increased vulnerability, independently or in interaction with these mechanisms, remains to be determined and warrants investigation in future longitudinal studies [44]. Especially in light of a recent study linking lipid profile alterations in ME/CFS to both circadian rhythm and sleep disturbances, with endothelial dysfunction proposed as an underlying mechanism [45].

Exploratory analyses did not reveal clear differences in estimated SE or variability across ME/CFS severity subgroups. These null findings should be interpreted cautiously. The severity-stratified analyses were underpowered and limited by a restricted severity range, as individuals with severe symptoms at rest were excluded. It is also plausible that individuals with more severe disease engage in stricter activity limitation to avoid symptom exacerbation, potentially stabilizing sleep patterns despite greater underlying pathology. Future studies incorporating larger samples across the full severity continuum, alongside concurrent assessment of daily symptoms and activity levels, are needed to clarify how disease severity modulates sleep variability.

Several limitations should be acknowledged. The sample size in subgroup analyses was relatively small, which may have limited our ability to detect more subtle effects. In addition, the exclusion of sleep disorders relied solely on interview-based medical history rather than systematic polysomnography. Consequently, the presence of undiagnosed sleep disorders cannot be entirely ruled out. The relatively short monitoring period also restricts the ability to characterize longer-term patterns, emphasizing the need for extended longitudinal assessments. Although accelerometry provides ecologically valid data, it does not capture sleep architecture or autonomic activity; combining multi-night ambulatory EEG with variability metrics would provide deeper mechanistic insight. Furthermore, daily symptom burden, activity levels, and PEM episodes were not modeled alongside variability, limiting the ability to directly link physiological triggers with sleep fluctuations.

Clinically, the present findings highlight that the frequent alternation between low- and relatively preserved estimated efficiency nights implies that the specific night selected for clinical evaluation may strongly influence diagnostic impressions and subsequent treatment decisions. Accordingly, extended multi-night assessment, ideally under ambulatory conditions (accelerometry, sleep diaries), may provide a more representative evaluation of sleep in this population. In addition, the coexistence of relatively regular sleep timing with increased estimated SE variability suggests that sleep fragmentation in ME/CFS may not primarily arise from irregular sleep scheduling, thereby distinguishing it from behavioral insomnia in which timing instability plays a central role. However, these observations also indicate that therapeutic approaches focusing solely on sleep duration or sleep opportunity may be insufficient, and that strategies aimed at reducing night-to-night fluctuation may warrant consideration in the clinical management of this population.

## Conclusion

This study demonstrates that people living with ME/CFS exhibited impaired sleep initiation and continuity, accompanied by pronounced night-to-night variability despite more stable sleep scheduling than controls. By integrating conventional sleep parameters with complementary variability metrics, we show that sleep in ME/CFS is highly variable. These findings underscore the importance of viewing variability as a central feature of ME/CFS-related sleep disturbances and point to the need to address the underlying physiological instability that may disrupt sleep-wake regulation in this condition.AQ

## Supplementary Information

Below is the link to the electronic supplementary material.

ESM 1 (DOCX 30.2 KB)

## Data Availability

The datasets supporting this analysis will be made available from the corresponding author upon reasonable request.

## Abbreviations

CFS: Chronic fatigue syndrome
COVID: Coronavirus disease
CV: Coefficient of variation
ESS: Epworth Sleepiness Scale
GGIR: Generic GitHub Implementation of Raw Accelerometer Analysis in R
ICC: International Consensus Criteria
IIV: Intraindividual variability
IOM: Institute of Medicine
IRB: Institutional Review Board
ME/CFS: Myalgic encephalomyelitis/chronic fatigue syndrome
PSG: Polysomnography
PSQI: Pittsburgh Sleep Quality Index
REM: Rapid eye movement
SARS-CoV-2: Severe acute respiratory syndrome coronavirus 2
SE: Sleep efficiency
SOL: Sleep onset latency
TIB: Time in bed
TST: Total sleep time
WASO: Wake after sleep onset
