# Supplementary material for: Sleep in myalgic encephalomyelitis/chronic fatigue syndrome shows marked night-to-night fluctuation under free-living conditions—results from a matched case-control study
Source: J Clin Sleep Med. 2026 May 13;22(1):77. doi: 10.1007/s44470-026-00079-7 (PMC13172179; doi:10.1007/s44470-026-00079-7)
Supplement: Supplementary file 1 — (DOCX 30.2 KB) [file 44470_2026_79_MOESM1_ESM.docx]

**Sleep in Myalgic Encephalomyelitis/Chronic Fatigue Syndrome Shows Marked Night-to-Night fluctuation under free-living conditions — Results from a Matched Case-Control Study**

Maïtena Saurel^1^, Isabelle Fornasieri^2,3^, Giovanna C. Del Sordo^4^, Cyril Chatain^1,5^, Livia Fantini^6^, Mathieu gruet^1^, Oussama Saidi^1*^

**Affiliation**

^1^ J-AP2S Laboratory, Toulon University, F-83041 Toulon, France.

^2^ ASFC, French ME/CFS Association, Nice, France.

^3^ Faculty of Psychology, University of Strasbourg, Strasbourg, France.

^4^ Psychology Department, New Mexico State University, 1780 E University Blvd, Las Cruces, NM 88003, USA

^5^ Inserm, CAPS UMR 1093, Burgundy Europe University, 21000 Dijon, France

^6^ Sleep Disorders Center, Neurophysiology Unit, Neurology Department, Clermont-Ferrand University Hospital, Université Clermont Auvergne, CNRS, Institut Pascal, 58,Rue Montalembert, 63000 Clermont-Ferrand, France

*** Address for Correspondence:**

Dr. Oussama SAIDI, professor (Associate)

Laboratory Youth-Physical Activity, Sport and Health (J-AP2S),

University of Toulon,

CS 60584, 83041 Toulon, Cedex 9,

**Email:** oussama.saidi@univ-tln.fr

**ORCID ID:** https://orcid.org/0000-0003-3005-8716

**Mobile:** +33 6 58 48 72 88

**Table S1.** Indices used to quantify intraindividual variability (IIV) of sleep parameters

| **Index** | **Conceptual focus** | **Formula** |
| --- | --- | --- |
| **Coefficient of Variation (CV)** | Global dispersion (magnitude of fluctuations scaled to mean level) | $CV=\frac{iSD}{iMean}\times100$ |
| **Root Mean Square of Successive Differences (RMSSD)** | between consecutive nights  Sequential variability (night-to-night variability) | $\mathrm{RMSSD} = \sqrt{\frac{1}{N-1} \sum_{i=1}^{N-1} {(x_{i+1}-x_{i})}^{2}}$ |
| **Bayesian Variability Model (BVM)** | Separation of systematic (latent) vs residual variability | $\mathrm{BVM} = Y_{\mathrm{ij}} \sim N\left( \mu_{j}, \sigma_{j} \right) \mathrm{where} \sigma_{j} \sim\text{Γ}\left( \alpha, \beta\right)$ |

IV, intraindividual variability; CV, coefficient of variation; RMSSD, root mean square of successive differences; BVM, Bayesian variability model; iSD, within-person standard deviation; iMean, within-person mean; N, number of nights; xᵢ, value at night i; μⱼ, participant-specific mean; σⱼ, participant-specific variability.

**Table S2.** Summary of comorbidities and treatments in participants ME/CFS and controls.

|  | **Controls** | **ME/CFS** |  |
| --- | --- | --- | --- |
| Comorbidities |  |  |  |
| *- Autoimmune / immune-related disorders* | 0 | 7 |  |
| *- Gastrointestinal disorders* | 0 | 3 |  |
| *- Endocrine / thyroid disorders* | 0 | 2 |  |
| *- Cardiometabolic disorders* | 4 | 5 |  |
| *- Allergies / MCAS* | 0 | 5 |  |
| *- Orthostatic Intolerance / POTS* | 0 | 21 |  |
| Treatments |  |  |  |
| *- Analgesics* | 1 | 5 |  |
| *- Neuropathic pain agents* | 0 | 11 |  |
| *- Beta-blockers* | 1 | 2 |  |
| *- Supplements* | 2 | 25 |  |
| *- Others* | 3 | 12 |  |

Data are expressed as number of participants; autoimmune and immune-related disorders include vitiligo, Sjögren’s syndrome, and fibromyalgia; gastrointestinal disorders include small intestinal bacterial overgrowth, chronic gastritis, and gastroparesis; allergic and mast cell–related disorders include conditions such as asthma and atopic dermatitis (eczema), as well as mast cell–mediated forms of angioedema; neuropathic pain agents include tricyclic antidepressants and serotonin and norepinephrine reuptake inhibitors used for chronic pain management; supplements include vitamins, minerals, and coenzyme Q10.

**Table S3.** Sensitivity analyses of habitual sleep and intraindividual variability adjusted for employment status

| Sleep Variable | Group | | | Employment Status | | |  |
| --- | --- | --- | --- | --- | --- | --- | --- |
|  | *t*-value | *β (95% CI)* | *p*-value | *t*-value | *β (95% CI)* | *p*-value |  |
| ***Means*** | | | | | | |  |
| Wake-up time (hh:mm ± hh:min) | 1.035 | 15.77 (-14.6; 46.15) | 0.304 | -1.135 | -17.46 (48.1; 13.19) | 0.260 |  |
| Bedtime (hh:mm ± hh:min) | -1.270 | -17.32 (-44.5; 9.87) | 0.208 | 0.649 | 8.92 (-18.5; 36.34) | 0.519 |  |
| TIB (min) | | 2.041 | 33.09 (0.77; 65.4) | **0.045** | -1.613 | -26.38 (-58.98; 6.22) | 0.111 |
| TST (min) | -0.384 | -4.55 (-28.14; 19.04) | 0.702 | -1.739 | -20.76 (-44.57; 3.04) | 0.086 |  |
| SE (%) | -7.400 | -5.81 (-7.37; -4.24) | **<0.001** | -0.008 | -0.01 (-1.58; 1.57) | 0.993 |  |
| SOL (min) | 4.286 | 12.23 (6.54; 17.92) | **<0.001** | -1.220 | -3.51 (-9.25; 2.22) | 0.226 |  |
| WASO (min) | 4.324 | 25.66 (13.83; 37.49) | **<0.001** | -0.378 | -2.26 (-14.19; 9.67) | 0.707 |  |
| ***CV*** | | | | | | |  |
| Wake-up time (%) | 1.123 | 1.23 (-0.96; 3.43) | 0.265 | 1.074 | 1.19 (-1.02; 3.4) | 0.286 |  |
| Bedtime (%) | -2.434 | -0.75 (-1.37; -0.14) | **0.017** | -0.601 | -0.19 (-0.81; 0.43) | 0.550 |  |
| TIB (%) | -1.235 | -1.48 (-3.86; 0.91) | 0.221 | 0.674 | 0.81 (-1.59; 3.22) | 0.503 |  |
| TST (%) | 2.990 | 3.46 (1.15; 5.76) | **0.004** | 1.402 | 1.64 (-0.69; 3.96) | 0.165 |  |
| SE (%) | 13.734 | 7.97 (6.82; 9.13) | **<0.001** | 1.449 | 0.85 (-0.32; 2.02) | 0.152 |  |
| SOL (%) | 0.279 | 1.94 (-11.94; 15.82) | 0.781 | 0.006 | 0.04 (-13.96; 14.04) | 0.995 |  |
| WASO (%) | 3.481 | 15.09 (6.45; 23.74) | **0.001** | 0.143 | 0.63 (-8.09; 9.35) | 0.886 |  |
| *RMSSD* | | | | | | |  |
| Wake-up time (%) | 2.560 | 20.98 (4.65; 37.32) | **0.013** | 0.127 | 1.05 (-15.43; 17.53) | 0.899 |  |
| Bedtime (%) | -1.481 | -10.63 (-24.93; 3.68) | 0.143 | 0.355 | 2.57 (-11.86; 17) | 0.724 |  |
| TIB (%) | -0.150 | -1.5 (-21.45, 18.46) | 0.882 | 0.094 | 0.95 (-19.18; 21.08) | 0.926 |  |
| TST (%) | 2.122 | 17.28 (1.05; 33.5) | **0.037** | 0.554 | 4.55 (-11.82; 20.92) | 0.582 |  |
| SE (%) | 10.666 | 8.41 (6.84; 9.99) | **<0.001** | 1.682 | 1.34 (-0.25; 2.92) | 0.097 |  |
| SOL (%) | 2.517 | 10.01 (2.08; 17.93) | **0.014** | -1.000 | -4.01 (-12; 3.99) | 0.321 |  |
| WASO (%) | 5.403 | 26.08 (16.46; 35.7) | **<0.001** | 1.233 | 6 (-3.7; 15.7) | 0.222 |  |
| *BVM* | | | | | | |  |
| Wake-up time (%) | 0.180 | 1.52 (-15.28; 18.32) | 0.858 | 0.022 | 0.19 (-16.76; 17.13) | 0.983 |  |
| Bedtime (%) | -2.468 | -6.07 (-10.97; -1.17) | **0.016** | -1.357 | -3.37 (-8.31; 1.58) | 0.179 |  |
| TIB (%) | -1.439 | -6.3 (-15.04; 2.43) | 0.155 | -1.158 | -5.12 (-13.93; 3.69) | 0.251 |  |
| TST (%) | -0.550 | -1.43 (-6.62; 3.76) | 0.584 | -0.111 | -0.29 (-5.53; 4.94) | 0.912 |  |
| SE (%) | -1.043 | -0.7 (-2.03; 0.64) | 0.300 | -1.720 | -1.16 (-2.5; 0.18) | 0.090 |  |
| SOL (%) | -1.610 | -3.57 (-7.99; 0.85) | 0.112 | -1.682 | -3.77 (-8.23; 0.7) | 0.097 |  |
| WASO (%) | -0.212 | -0.58 (-6.06; 4.9) | 0.833 | -1.634 | -4.53 (-10.06; 1) | 0.107 |  |

Values are t-statistics and p-values from linear regression models including Group (ME/CFS vs controls) and Employment status (employed vs non-employed) as predictors. “Group” reflects the adjusted case–control effect; “Employment status” reflects its independent association with each sleep outcome. CV: coefficient of variation; RMSSD: root mean square of successive differences; BVM: Bayesian variability model. Bold indicates p < 0.05.

STROBE Statement—Checklist of items that should be included in reports of ***case-control studies***

|  | Item No | Recommendation | Page No |
| --- | --- | --- | --- |
| **Title and abstract** | 1 | (*a*) Indicate the study’s design with a commonly used term in the title or the abstract | 1 |
|  |  | (*b*) Provide in the abstract an informative and balanced summary of what was done and what was found | 4 |
| Introduction | | | |
| Background/rationale | 2 | Explain the scientific background and rationale for the investigation being reported | 6-7 |
| Objectives | 3 | State specific objectives, including any prespecified hypotheses | 7-8 |
| Methods | | | |
| Study design | 4 | Present key elements of study design early in the paper | 8 |
| Setting | 5 | Describe the setting, locations, and relevant dates, including periods of recruitment, exposure, follow-up, and data collection | 8 |
| Participants | 6 | (*a*) Give the eligibility criteria, and the sources and methods of case ascertainment and control selection. Give the rationale for the choice of cases and controls | 8-9 |
|  |  | (*b*) For matched studies, give matching criteria and the number of controls per case | 8-9 |
| Variables | 7 | Clearly define all outcomes, exposures, predictors, potential confounders, and effect modifiers. Give diagnostic criteria, if applicable | 8-12 |
| Data sources/ measurement | 8* | For each variable of interest, give sources of data and details of methods of assessment (measurement). Describe comparability of assessment methods if there is more than one group | 8-12 |
| Bias | 9 | Describe any efforts to address potential sources of bias | 8-9 |
| Study size | 10 | Explain how the study size was arrived at | 12 |
| Quantitative variables | 11 | Explain how quantitative variables were handled in the analyses. If applicable, describe which groupings were chosen and why | 13 |
| Statistical methods | 12 | (*a*) Describe all statistical methods, including those used to control for confounding | 13 |
|  |  | (*b*) Describe any methods used to examine subgroups and interactions | 13 |
|  |  | (*c*) Explain how missing data were addressed | 13 |
|  |  | (*d*) If applicable, explain how matching of cases and controls was addressed | 12 |
|  |  | (*e*) Describe any sensitivity analyses | 13 |
| Results | | | |
| Participants | 13* | (a) Report numbers of individuals at each stage of study—eg numbers potentially eligible, examined for eligibility, confirmed eligible, included in the study, completing follow-up, and analysed | N/A |
|  |  | (b) Give reasons for non-participation at each stage | N/A |
|  |  | (c) Consider use of a flow diagram | N/A |
| Descriptive data | 14* | (a) Give characteristics of study participants (eg demographic, clinical, social) and information on exposures and potential confounders | 13-14 |
|  |  | (b) Indicate number of participants with missing data for each variable of interest | 10-11 |
| Outcome data | 15* | Report numbers in each exposure category, or summary measures of exposure | 9 |

| Main results | | 16 | (*a*) Give unadjusted estimates and, if applicable, confounder-adjusted estimates and their precision (eg, 95% confidence interval). Make clear which confounders were adjusted for and why they were included | N/A |
| --- | --- | --- | --- | --- |
|  |  |  | (*b*) Report category boundaries when continuous variables were categorized | N/A |
|  |  |  | (*c*) If relevant, consider translating estimates of relative risk into absolute risk for a meaningful time period | N/A |
| Other analyses | 17 | Report other analyses done—eg analyses of subgroups and interactions, and sensitivity analyses | | 14-16 |
| Discussion | | | | |
| Key results | 18 | Summarise key results with reference to study objectives | | 17 |
| Limitations | 19 | Discuss limitations of the study, taking into account sources of potential bias or imprecision. Discuss both direction and magnitude of any potential bias | | 20 |
| Interpretation | 20 | Give a cautious overall interpretation of results considering objectives, limitations, multiplicity of analyses, results from similar studies, and other relevant evidence | | 20-21 |
| Generalisability | 21 | Discuss the generalisability (external validity) of the study results | | 20-21 |
| Other information | | | | |
| Funding | 22 | Give the source of funding and the role of the funders for the present study and, if applicable, for the original study on which the present article is based | | 2 |
